# Non-acid reflux and reflux laryngitis: new perspectives on mechanism, diagnosis and treatment

**DOI:** 10.3389/fmed.2026.1949945

**Published:** 2026-10-12

**Authors:** Hongshan Chang, Rui Sun, Huan Qiu, Yunhong Ning, Lei Yao

**Affiliations:** 1 First College of Clinical Medicine, Shandong University of Traditional Chinese Medicine, Jinan, China; 2 Department of Ultrasound Medicine, Affiliated Hospital of Xuzhou Medical University, Xuzhou, China; 3 Department of Otolaryngology, Affiliated Hospital of Shandong University of Traditional Chinese Medicine, Jinan, China

**Keywords:** diagnosis, mechanism of action, non-acid reflux, reflux laryngitis, treatment

## Abstract

Non-acid reflux may serve as a potential contributing factor in the pathogenesis of reflux pharyngitis. Its plausible injury-related mechanisms may partly explain difficult-to-treat clinical presentations and contribute to diagnostic and therapeutic challenges, with consequent impacts on patients' quality of life. Recently, the potentially significant role of non-acid reflux in refractory reflux pharyngitis has emerged as a prominent topic in this field, unveiling a novel pathophysiological mechanism that transcends the traditional gastric acid damage model. This underlying mechanism encompasses the independent and synergistic damaging effects of various active components, including bile acids, pancreatic enzymes, pepsin and gas reflux. It involves multiple pathways, such as intracellular reactivation, disruption of tight junctions, inflammatory cascade responses, and hypoxic injury. This article provides a narrative review discussing potential roles of non-acid reflux in the pathogenesis of reflux pharyngitis, focusing on elucidating the pathogenic mechanisms associated with each component of non-acid reflux, the advancements and limitations of current diagnostic techniques, and comprehensive intervention strategies. This review may hold potential clinical translational value for optimizing the hierarchical management of reflux pharyngitis and alleviating the symptom burden in refractory patients.

## 1 Highlights

- Evidence synthesis suggests non-acid reflux as a potential factor in refractory LPR;
- Integrates NF-*κ*B, MAPK and multiple inflammatory pathways into a network;
- Highlights MII-pH's diagnostic blind spots for non-acid reflux events;
- Proposes a multi-intervention framework: physical barriers, mucosal protection, and component antagonism.

## 2 Search strategy

This narrative review, distinct from a systematic review, identifies relevant literature through a search of the PubMed and Web of Science databases up to August 1, 2026. We employed the following Boolean search strategy: (“laryngopharyngeal reflux” OR “LPR” OR “reflux laryngitis”) AND (“non-acid reflux” OR “bile acids” OR “pepsin” OR “trypsin” OR “pancreatic enzymes” OR “gas reflux”) AND (“mechanism” OR “inflammation” OR “diagnosis” OR “treatment” OR “therapy”). Our search was limited to English-language publications, encompassing original research, reviews, and clinical studies, without restricting the publication date to ensure comprehensive coverage of both foundational and recent findings. Additionally, we manually screened the reference lists of identified articles to capture further relevant studies. Studies were included if they addressed mechanisms, diagnostic approaches, or therapeutic strategies related to non-acid reflux within the context of laryngopharyngeal reflux or closely related upper aerodigestive tract disorders. We excluded conference abstracts, case reports, and non-English publications.

**Figure 1:**
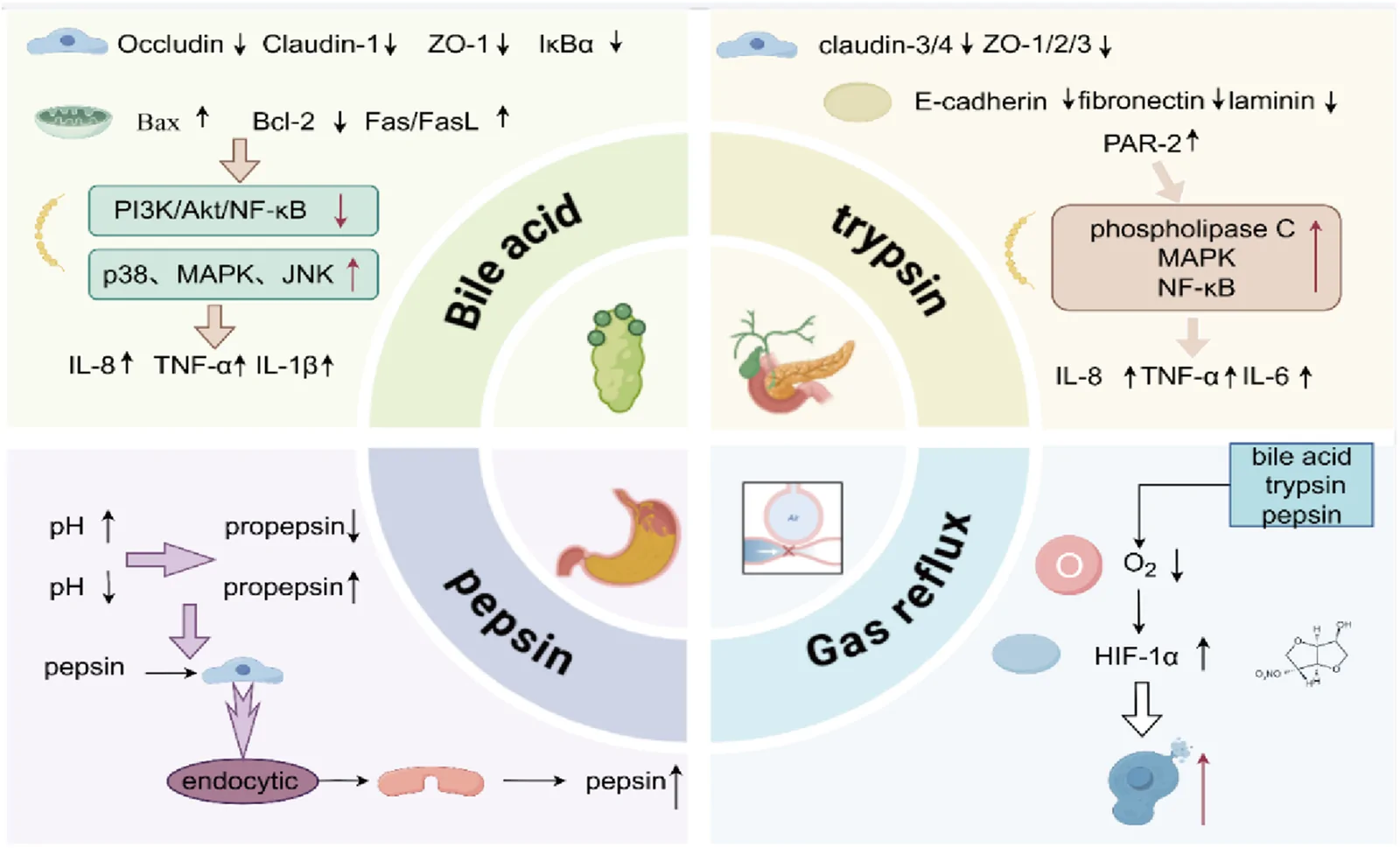
Non-acid reflux-induced mucosal injury pathways. All arrows in this figure represent mechanisms that are predominantly based on indirect evidence from *in vitro* studies, animal models, or other diseases.

**Table 1:** Pathogenic mechanisms of the main active ingredients for non-acid reflux.

Ingredients	Primary mechanism of action	Key molecules/pathways	Ref.
Bile acids	Disruption of cell membrane integrity and tight junctions; oxidative stress and inflammatory activation; induction of apoptosis; enhanced pH-dependent toxicity	ROS, NF-*κ*B, MAPK, caspase-3/8/9, Bax/Bcl-2	(19, 27, 31, 37, 46)
Trypsin	Directly hydrolyzes tight junction proteins and E-cadherin; activates the PAR-2-mediated inflammatory cascade	claudin-3/4, ZO, PAR-2, PLC, MAPK, NF-κB	(60, 61, 63)
Pepsin	pH-dependent activation/reactivation; reactivation following endocytosis	Acidic organelles	(79, 81)
Reflux gas	Overcoming the UES barrier to deliver aerosolized active substances; reflux-associated hypoxia activates HIF-1*α*	UES, aerosol, HIF-1*α*	(86, 94)

ROS, reactive oxygen species; NF-κB, nuclear factor-kappa B; MAPK, mitogen-activated protein kinase; PAR-2, protease-activated receptor 2; PLC, phospholipase C; UES, upper esophageal sphincter; HIF-1α, hypoxia-inducible factor-1α. Level of Evidence: All mechanisms listed in the table are primarily indirect; there may currently be a lack of direct evidence regarding LPR.

**Table 2:** Major diagnostic techniques for reflux pharyngitis and their advantages and disadvantages.

Diagnostic Techniques	Advantages	Limitations	Ref.
RSI/RFS	Simple, non-invasive, and widely used in clinical practice	Symptoms and signs are nonspecific; they cannot be used to confirm a diagnosis.	(97, 99)
24-hour esophageal pH monitoring	Objective and reproducible; quantifies acid reflux burden	Can only detect acid reflux	(100, 101)
MII-pH	Widely used monitoring tool; identifies acid, weak acid, and non-acid reflux	Highly invasive; expensive equipment; complex operation	(102, 103, 106)
Dx-pH Monitoring System	Simple and minimally invasive; specifically designed for LPR	The diagnostic and therapeutic value remains controversial	(107, 109)
96-Hour Wireless pH Monitoring	Extended monitoring time; no nasopharyngeal discomfort; closer to physiological conditions	Can only detect acid reflux; high equipment costs; requires endoscopic procedures	(110, 112)
Salivary Pepsin Test	Non-invasive, simple, fast, and reproducible; highly sensitive	Low specificity; unable to distinguish between acid reflux and non-acid reflux	(113, 115)

LPR, laryngopharyngeal reflux; RSI, reflux symptom index; RFS, reflux finding score; MII-pH, multichannel intraluminal impedance-pH monitoring. Current Controversy: There are currently no universally accepted diagnostic criteria for non-acid reflux. The clinical utility of each diagnostic technique may still be a matter of debate.

**Table 3:** Treatment strategies for non-acid reflux.

Treatment Strategies	Mechanism of action	Notes	Ref.
UDCA	Displaces and inhibits hydrophobic bile acids; stabilizes cell membranes; reduces oxidative stress and NF-κB-mediated inflammatory activation	For bile acid reflux; has a cell-protective effect in Barrett's esophagus	(120)
Aginate	Forms a low-density gel raft in the acidic environment of the stomach, acting as a pH-independent physical barrier to block refluxed material including non-acid components	Relieve LPR-related symptoms	(122, 124, 125)
Sucralfate	Forms a protective gel film on damaged pharyngeal mucosa, physically isolating mucosa from pepsin and bile acids in refluxate	Mucosal protectant; does not rely on acid suppression	(119, 126)
Pepsin inhibitors	Directly inhibits pepsin activity, blocking its proteolytic and inflammatory effects at the source	May be considered a potential treatment option; clinical application remains investigational	(127)

LPR, laryngopharyngeal reflux; UDCA, ursodeoxycholic acid; MII-pH, multichannel intraluminal impedance-pH monitoring; PPI, proton pump inhibitor; GERD, gastroesophageal reflux disease. All treatment strategies listed in the table are based on indirect, experimental, or preclinical evidence.

**Table 4:** Summary of evidence for key mechanistic and therapeutic claims.

Core claim	Evidence Source	Evidence category	Ref.
Bile acids disrupt tight junctions and activate NF-κB/MAPK inflammatory pathways	Pharyngeal-derived cell lines; GERD/Barrett's-esophagus animal models	Indirect evidence: *in-vitro* + animal-model data	(25) (26) (34) (36)
Bile acids induce apoptosis and pH-dependent cytotoxicity	Cancer-derived and epithelial cell culture systems	Indirect evidence: *in-vitro* data	(43) (50)
Trypsin hydrolyzes claudin-3/4 and E-cadherin; activates PAR-2 inflammatory cascade	Rat esophagitis models; gastrointestinal epithelial cell experiments	Indirect evidence: animal-model + *in-vitro* data	(60) (61) (63)
Pepsin is endocytosed and re-activated intracellularly	Human laryngeal epithelial cell *in-vitro* confocal assays	Indirect evidence: *in-vitro* data	(81) (82)
Gas reflux overcomes UES and delivers aerosolized substances; hypoxia activates HIF-1α	Physiological monitoring studies; hypoxia-cell-culture models	Indirect/observational inferential evidence	(86) (94)
UDCA reduces bile-acid-mediated mucosal injury	Patients with bile-reflux gastritis/Barrett's esophagus	Indirect human evidence	(120) (121)
Alginate and sucralfate form physical mucosal-protective barriers	*In-vitro* barrier models; clinical cohorts of GERD/LPR-symptom patients	Mixed *in-vitro* plus observational clinical evidence	(122) (123) (124)
Pepsin inhibitors reduce reflux-associated mucosal inflammation	GERD mouse-model experiments	Pre-clinical animal-model evidence	(128) (129)

LPR, laryngopharyngeal reflux; UES, upper esophageal sphincter; HIF-1α, hypoxia-inducible factor-1α; UDCA, ursodeoxycholic acid; GERD, gastroesophageal reflux disease. Relevance and Level of Evidence: All of the arguments listed are supported solely by indirect evidence; there is currently no direct evidence from patients with LPR.

## 3 Introduction

Laryngopharyngeal reflux (LPR) is a clinical syndrome characterized by the abnormal reflux of gastric and duodenal contents into the pharynx, leading to damage of the mucous membranes in the affected area. This condition results in a range of chronic inflammatory symptoms and signs. Common clinical manifestations include hoarseness, vocal fatigue, frequent throat clearing, chronic dry cough, a sensation of a foreign body in the pharynx (globus hystericus), and excessive mucus production (1, 2). LPR is a prevalent condition encountered in the outpatient otolaryngology department. Approximately 10% of patients seeking care in this specialty present with symptoms directly associated with LPR, while the incidence among individuals with voice disorders, such as hoarseness, may exceed 50% (3, 4). However, the non-specific nature of the symptoms, coupled with the limitations of diagnostic methods, has resulted in substantial economic and psychological burdens on patients (5). Consequently, conducting in-depth research on the pathogenesis of LPR, as well as optimizing diagnostic and treatment strategies, holds considerable clinical significance and social value for enhancing the prognosis of numerous patients.

The conventional perspective posits that the primary pathological mechanism of LPR is the direct chemical injury inflicted by gastric acid on the pharyngeal mucosa (6–8). Consequently, effective acid suppression therapy, particularly with proton pump inhibitor (PPI), has been widely considered the cornerstone of LPR management (9). Gastroesophageal reflux disease (GERD) and LPR are both part of the reflux disease spectrum, exhibiting a close pathophysiological relationship (10). However, clinical observations indicate that some patients with LPR do not adequately respond to standard or double-dose PPI therapy, often continuing to experience persistent symptoms. This phenomenon poses a significant challenge in clinical diagnosis and treatment (11, 12). This observation suggests that inhibition of gastric acid alone may not fully block the pathogenic process of LPR, and non-acidic reflux constituents may also contribute to reflux-related pharyngeal injury. Consequently, there is an urgent need to re-evaluate other potential pathogenic factors and treatment modalities beyond gastric acid.

Non-acidic reflux pertains to the pH level of the reflux material. Nevertheless, the presence of bile acids, pancreatic enzymes, pepsin and certain gases can still inflict ongoing and cumulative harm to the pharyngeal mucosa (13). Pepsin exhibits stability in non-acidic environments and can adhere to pharyngeal epithelial cells. Upon re-exposure to acidic substances, pepsin becomes activated, resulting in a cycle of recurrent mucosal damage that does not depend on ongoing acid exposure (14, 15).

This review elucidates the potential mechanisms underlying non-acid reflux and summarizes its possible role in LPR. Furthermore, this review synthesizes potential diagnostic techniques and treatment strategies, moving beyond the limitations of the traditional focus on gastric acid suppression alone. We hope this review will provide clinicians with preliminary insights for future research, while acknowledging that definitive causal relationships and treatment efficacy still require further validation.

## 4 Components and mechanism of action of non-acid reflux

Mucosal damage resulting from LPR is not exclusively attributed to gastric acid. Non-acid reflux (NAR) components, including bile acids, pancreatic enzymes, pepsin and gas, may also play a significant role in causing the disease (16, 17). This chapter describes the primary active substances that comprise NAR and their associated pathogenic mechanisms. It aims to unveil the theoretical foundation of the pathological essence of LPR, thereby providing a scientific basis for future investigations into precise diagnostic methods and targeted intervention strategies. It is important to note that the components of reflux discussed below—bile acids, pancreatic enzymes, pepsin, and gas—belong to different conceptual categories: bile acids and pancreatic enzymes are digestive factors originating in the duodenum; pepsin is a protease originating in the stomach; and gas reflux refers to a physical state rather than a chemical composition. These components are not unique to non-acid reflux; they may also coexist with acid or weakly acidic reflux. This distinction is crucial for a correct understanding of the pathogenic mechanisms and treatment strategies discussed in subsequent chapters. (as shown in Figure 1 and Table 1).

### 4.1 Bile acid

Bile acids are the primary solid and active constituents of bile, classified as amphiphilic steroid compounds synthesized from cholesterol in the liver (18). They play a crucial role in the digestion and absorption of lipids, as well as in the regulation of cholesterol homeostasis (19). Bile acid, recognized as the most representative harmful component in duodenal content reflux (20), exhibits multi-target characteristics in its detrimental effects on the pharyngeal mucosa. This damage occurs independently of the corrosive effects associated with gastric acid (21).

#### 4.1.1 Cell membrane destruction and barrier dysfunction

Evidence regarding bile acid-induced disruption of epithelial barrier function in LPR is entirely indirect. No direct observation of this mechanism in LPR pharyngeal mucosa has been reported. *In vitro* studies have shown that, bile acids, characterized by their amphiphilic molecular structure, can infiltrate the lipid bilayer of pharyngeal epithelial cells, thereby enhancing cell membrane permeability and resulting in mitochondrial dysfunction and ATP depletion (22–24). In animal models of reflux esophagitis, bile acids have been shown to down-regulate tight junction proteins including occludin, claudin-1, and ZO-1, or to induce their redistribution (25, 26). The disruption of tight junctions enhances epithelial permeability, facilitating the penetration of macromolecular substances from the refluxate through the epithelial barrier and into the submucosa, thereby triggering inflammatory responses and activating nerve endings (27, 28). Based on these indirect observations, it has been hypothesized that similar mechanisms might operate in LPR, potentially establishing a “damage-increased permeability-further damage” cycle in the pharyngeal mucosa.

#### 4.1.2 Oxidative stress and activation of inflammatory responses

Evidence supporting these inflammatory signaling observations is mainly derived from *in-vitro* human epithelial-cell experiments. Direct evidence obtained from laryngopharyngeal mucosal biopsies of LPR patients remains scarce. Therefore, descriptions below represent mechanistic hypotheses instead of confirmed pathological events occurring in human LPR.

*In vitro* experiments using human pharyngeal epithelial cells have shown that upon entering pharyngeal epithelial cells, bile acids can trigger an excessive production of reactive oxygen species (ROS), primarily due to the dysfunction of mitochondrial respiratory chain activity and the activation of NADPH oxidase (29, 30). This overproduction of ROS not only directly leads to lipid peroxidation, protein carbonylation, and oxidative damage to DNA, but more significantly, it serves as a second messenger that activates various inflammation-related signaling pathways (31, 32).

The activation of the nuclear factor *κ*B (NF-*κ*B) pathway plays a crucial role in reflux diseases (33). ROS can promote the phosphorylation of I*κ*B kinase (IKK), leading to the degradation of the NF-*κ*B inhibitor protein (I*κ*B*α*). This mechanism facilitates the nuclear translocation of NF-*κ*B, thereby initiating the transcription of downstream pro-inflammatory cytokines (34, 35). Studies have found that treatment with deoxycholic acid significantly upregulates the mRNA expression levels of IL-8, TNF-α, and IL-1β in human pharyngeal epithelial cells. Notably, this effect can be inhibited by antioxidant pretreatment (36). It should be emphasized once again that the evidence supporting the activation of the aforementioned inflammatory pathways comes primarily from *in vitro* experiments, while direct validation in human pharyngeal mucosal tissue under *in vivo* conditions is notably lacking. Therefore, the true pathological role of these mechanisms in patients with LPR requires further study.

Based on research findings regarding other inflammatory diseases, such as liver fibrosis and lung diseases, bile acids can activate the MAPK pathway, including p38 MAPK and JNK, thereby promoting the release of inflammatory mediators (37, 38). In other inflammatory disease models, these inflammatory factors recruit neutrophils and macrophages to local tissues and activate fibroblasts and epithelial cells, creating a positive feedback loop that results in persistent inflammation and chronic tissue damage (39). Although there are currently no similar studies specifically targeting the pharyngeal mucosa, given that pharyngeal epithelial cells may also possess the potential for inflammatory activation, it is speculated that this mechanism may also apply to the pathogenesis of reflux pharyngitis. The maturation of dendritic cells, the differentiation of Th1/Th17 T cells, and the recruitment of macrophages have also been observed in other chronic inflammatory diseases of the upper gastrointestinal tract and respiratory tract (40–42). Based on this, it can be inferred that non-acid reflux could, in theory, trigger a similar shift from innate to adaptive immunity in the pharyngeal mucosa. However, whether this mechanism actually exists in chronic LPR remains purely hypothetical. There's a lack of direct evidence from throat tissues of LPR patients, so future studies need to figure out whether adaptive immune responses are involved in the chronic development of LPR.

#### 4.1.3 Induction of apoptosis

Evidence for bile acid-induced apoptosis in LPR mucosa is derived exclusively from *in vitro* and cancer-related studies; direct evidence in LPR patients is absent. In cultured cell systems, high concentrations of bile acids can induce apoptosis via two primary pathways. The first pathway is the death receptor pathway, which activates caspase-8 through the upregulation of Fas/FasL expression. The second pathway is the mitochondrial pathway, which releases cytochrome C by inducing mitochondrial permeability transition and subsequently activates caspase-9. Ultimately, both pathways converge to activate the effector caspase-3, thereby initiating the apoptotic program (43–45).

Studies using the T24 bladder cancer cell line have demonstrated that bile acids inhibit cell proliferation and promote apoptosis by inducing oxidative stress-mediated mitochondrial dysfunction and by inhibiting the PI3 K/Akt/NF-*κ*B signaling pathway (46). Extending these apoptotic effects to normal pharyngeal epithelial cells in an LPR context suggests that they may produce similar effects; however, this remains merely a mechanistic hypothesis at this time. From a similar perspective, it is evident that high concentrations of bile acids can induce apoptosis via the mitochondrial pathway by activating the p53 signaling pathway, which leads to the upregulation of the pro-apoptotic protein Bax and the downregulation of the anti-apoptotic protein Bcl-2.

Persistent apoptosis and an imbalance in compensatory proliferation may compromise epithelial barrier function (47). Furthermore, the accumulation of DNA damage resulting from repeated episodes of apoptosis and regeneration, coupled with oxidative stress in the inflammatory microenvironment, may establish a basis for atypical hyperplasia or even carcinogenesis of the laryngeal mucosa (48, 49). However, these are extrapolative hypotheses derived from non-LPR cell systems and cancer models; no direct evidence links bile acid-induced apoptosis to LPR pathology in patients.

#### 4.1.4 Enhanced PH-dependent cytotoxicity

The cytotoxic effects of bile acids exhibit a pronounced pH-dependent characteristic. In acidic and weakly acidic microenvironments, undissociated bile acids, which exist in molecular form, demonstrate increased liposolubility, allowing them to efficiently penetrate the cell membrane and enter the intracellular space via passive transmembrane diffusion (50, 51); Once inside the cell, these acids dissociate under near-neutral pH conditions, remaining in ionic form and accumulating, which mediates cellular damage and toxic effects (19, 52). This mechanism indicates that although the overall pH value of gastroesophageal reflux is higher than 4.0, the transient acidic microenvironment on the local mucosal surface, caused by external factors such as the intake of acidic beverages, can significantly enhance the transmembrane transport efficiency and intracellular retention rate of bile acids. This enhancement effect, in turn, will exacerbate its harmful effects on the mucosal epithelium (53).

In conclusion, findings from *in-vitro* cell experiments and animal reflux models suggest that bile acids may exert damaging effects originating from duodenal reflux, with plausible multi-target injurious actions on pharyngeal mucosa independent of gastric-acid-mediated injury. Based on these indirect data, bile acids may disrupt epithelial lipid bilayers and alter tight-junction protein expression and distribution, potentially creating a self-reinforcing cycle of mucosal injury and increased permeability.

After cellular uptake, bile acids may trigger mitochondrial dysfunction and oxidative-stress responses and may activate NF-*κ*B and MAPK-dependent inflammatory cascades to up-regulate pro-inflammatory mediators including IL-8 and TNF-*α*. *In-vitro* data also indicate that high bile-acid concentrations may promote epithelial apoptosis through death-receptor and mitochondrial-related pathways, which could disturb epithelial cell turnover and barrier integrity. Repeated injury-repair cycles together with oxidative stress might theoretically raise the hypothetical risk of laryngeal mucosal atypical hyperplasia. Bile-acid cytotoxicity appears pH-dependent in experimental systems, where acidic microenvironments favor intracellular bile-acid accumulation and cell injury.

### 4.2 Trypsin

Trypsin, a serine protease secreted by the pancreas (54), may travel retrograde with reflux into the pharynx during duodenogastroesophageal reflux, where it exerts a proteolytic effect on the mucosal barrier (55). The pharyngeal mucosal barrier serves as a physical defense structure composed of epithelial cells and intercellular junction complexes, and its integrity is essential for resisting the invasion of harmful substances present in reflux (56). But, the presence and pathogenic role of trypsin in LPR pharyngeal mucosa have not been directly demonstrated in patients. The following mechanisms are derived from animal models and should be interpreted as hypotheses requiring LPR-specific validation.

#### 4.2.1 The hydrolysis of tight junction proteins by trypsin

Tight junctions are intercellular junction complexes that consist of transmembrane proteins, junction adhesion molecules (JAMs), and intracellular scaffold proteins, including zonula occludens (ZO-1, ZO-2, ZO-3). These structures are situated on the apical surface of epithelial cells and serve a critical role in sealing the intercellular space while regulating transcellular transport of substances (57, 58). As a serine protease, trypsin can specifically hydrolyze the peptide bonds within tight junction proteins, resulting in the disruption of the tight junction complex and the subsequent loss of barrier function (59).

In animal models of reflux esophagitis, it has been observed that trypsin hydrolyzes tight junction proteins. Research utilizing rat esophagitis models has observed that trypsin treatment results in the down-regulation of claudin-3 and claudin-4 expression while simultaneously cleaving cell adhesion molecules such as E-cadherin, thereby disrupting the junctional architecture between epithelial cells (60, 61). Furthermore, trypsin is capable of degrading extracellular matrix components, including fibronectin and laminin, which further compromises the integrity of the mucosal structure. This degradative capacity exhibits certain similarities to that of chymase, another member of the serine protease family (62).

These observations in animal models have led to the hypothesis that trypsin might similarly disrupt intercellular junctions in LPR, potentially leading to widened intercellular spaces and increased mucosal permeability.

#### 4.2.2 Inflammatory cascade: an indirect mechanism by which pancreatic enzymes activate immune responses

In addition to its direct hydrolysis of the mucosal barrier, trypsin can initiate an inflammatory cascade by activating protease-activated receptors (PARs) on the cell surface. But, the proposed trypsin-mediated inflammatory cascade in LPR is entirely based on indirect evidence from other systems (63).

PARs are members of the G protein-coupled receptor family. Currently, four members have been identified (PAR-1 to PAR-4), with PAR-2 serving as the primary target of trypsin (64, 65). PARs exhibit a distinctive activation mechanism: trypsin cleaves the extracellular N-terminal region of the receptor, thereby exposing a new N-terminal sequence that functions as a “ligand” to bind to the receptor itself. This interaction triggers conformational changes and initiates intracellular signal transduction (66, 67). Studies have observed that trypsin specifically cleaves the N-terminal domain of PARs at the R36 S37 site, releasing a new N-terminal sequence that acts as an autoligand, termed the “lacing ligand,” which binds to the second extracellular loop of the receptor (68). This irreversible proteolytic activation mechanism allows PARs to transform transient protease exposure into sustained cellular signaling events.

Following the activation of PAR-2, several inflammation-related pathways can be initiated: it activates phospholipase C, leading to the release of intracellular calcium ions (69); it activates the MAPK pathway (70); it activates the NF-*κ*B pathway (71). The activation of these signaling pathways ultimately enhances the transcription and release of pro-inflammatory cytokines (72).

The activation of the aforementioned signaling pathways ultimately results in the transcription and release of various pro-inflammatory cytokines. Studies have demonstrated that treatment of human esophageal epithelial cells with trypsin or a PAR-2 agonist peptide significantly increases the mRNA expression and protein secretion levels of IL-8 in a dose-dependent manner (63). Additionally, PAR activation promotes the production of several pro-inflammatory cytokines, including IL-6 (40), indicating its extensive role in the inflammatory cascade response. IL-8, a neutrophil chemokine, recruits inflammatory cells to local tissues (41); IL-6 participates in B cell differentiation and acute-phase protein synthesis (73); and TNF-α, a key pro-inflammatory mediator, further amplifies the inflammatory response (42). The synergistic effects of these cytokines create a positive feedback inflammatory cycle, contributing to the persistence of tissue damage. In human respiratory epithelial cells, PAR-2 activation has been shown to stimulate IL-6 and IL-8 release and has been associated with regulation of antigen-presenting capacity (40, 66). Although these findings derive from models of allergic airway inflammation rather than reflux-related mucosal injury, they raise the hypothetical possibility that trypsin might exert immunomodulatory effects beyond innate inflammation via PAR-2 activation in pharyngeal epithelium. Whether this actually occurs in LPR remains entirely speculative and awaits direct investigation in LPR-specific models.

In conclusion, observations obtained from animal esophagitis models and *in-vitro* epithelial-cell systems suggest that trypsin may disrupt pharyngeal mucosal barrier function via two hypothetical pathways. Direct proteolytic activity may degrade tight-junction proteins (claudin-3/-4), E-cadherin and extracellular-matrix constituents, potentially widening intercellular spaces and increasing epithelial permeability. Indirectly, trypsin-driven PAR-2 cleavage ay initiate phospholipase-C-calcium, MAPK and NF-*κ*B downstream signaling, promoting pro-inflammatory mediators (IL-6, IL-8, TNF-*α*) and inflammatory-cell recruitment, possibly forming self-amplifying inflammatory loops.

Detecting trypsin activity in saliva or pharyngeal secretions may serve as a potential investigational biomarker for assessing non-acid reflux exposure and potential mucosal injury (74). However, this approach remains investigational; no clinical validation studies in LPR patients have yet established its diagnostic utility.

### 4.3 Pepsin

Pepsin, an aspartic acid protease, is secreted by the principal cells of the gastric mucosa. It is activated from its inactive form, pepsinogen, in an acidic environment (pH < 5) and serves as the primary proteolytic enzyme in gastric juice (75–77). In the pathological process of LPR, pepsin is typically considered one of the non-acid reflux components that contribute to tissue damage (78). While it shares some pathogenic mechanisms with bile acids and pancreatic enzymes, pepsin possesses distinct characteristics that render it one of the key factors that cannot be overlooked in the pathophysiology of refractory LPR. However, the direct pathogenic mechanisms of pepsin in LPR pharyngeal tissue remain incompletely understood, with most mechanistic evidence derived from *in vitro* systems.

#### 4.3.1 Pepsin Ph-dependent

The activity of pepsin is closely linked to its pH environment (79). The enzyme exhibits optimal catalytic activity at a pH of approximately 2.0. As the pH increases, its proteolytic capability gradually diminishes, and catalytic activity is essentially lost at a pH of around 7–8 (79, 80). However, the structural stability of pepsin can be maintained across a broader pH range. Even in a pH 8.0 environment, the enzyme retains its spatial conformation and can be reactivated upon a subsequent decrease in pH (14, 80). This indicates that after pepsin enters the throat during reflux, it can still be reactivated, even if neutralized to a non-acidic environment by saliva and local bicarbonate, particularly if an acidic diet is consumed thereafter, leading to ongoing mucosal damage. But the relevance of these pH-dependent properties to pharyngeal mucosal injury in LPR patients has not been directly demonstrated.

#### 4.3.2 Intracellular mechanism of pepsin action

The traditional perspective posits that pepsin exerts its proteolytic effects solely in the extracellular environment. However, this extracellular pH dependence represents only one facet of pepsin's pathogenic mechanism. Recent studies indicate that pepsin can also penetrate pharyngeal epithelial cells via the endocytosis pathway (81, 82). Once inside the cell, pepsin can regain its enzymatic activity within acidic organelles, such as endosomes and the Golgi apparatus, where the pH is lower. This activation disrupts the homeostasis of intracellular proteins and triggers a cellular stress response (81–84). Consequently, this intracellular activation mechanism allows pepsin to inflict ongoing cellular damage, even in a neutrophilic extracellular environment, which markedly contrasts with the extracellular action mechanisms of bile acids and pancreatic enzymes.

In conclusion, experimental data indicate that pepsin bio-activity is highly pH-dependent. *In-vitro* confocal imaging supports the hypothetical model that pepsin may gain intracellular access through endocytosis and regain bio-activity inside acidic intracellular organelles, which might disturb cellular homeostasis. Currently, direct evidence indicating that pepsin enters pharyngeal epithelial cells through endocytosis is largely confined to *in vitro* confocal imaging. Further validation is necessary using animal models or human pharyngeal biopsy specimens. Until such validation is achieved, this should be regarded as a hypothesis requiring further translational investigation, rather than an established pathogenic mechanism in LPR.

### 4.4 Gas reflux

Gas reflux contributes to the pathogenesis of laryngeal reflux (85). Unlike liquid reflux, gas reflux is more adept at breaching the mechanical barrier of the upper esophageal sphincter (UES), allowing the transport of active components to more distant anatomical sites (86, 87). This process plays a significant role in the onset and progression of mucosal injury in the pharynx.

The UES serves as the final barrier against reflux in the pharynx, employing a selective opening mechanism that depends on the physical state of the reflux (88). Research indicates that liquid reflux necessitates adequate volume and pressure to surpass the tension of the UES, whereas gas reflux can easily traverse when the sphincter briefly relaxes (89). Consequently, the resistance of the UES to gas is significantly lower than that to liquid, allowing even minor pressure fluctuations to induce gas backflow events. Furthermore, gas reflux can disseminate active ingredients over a broader area of the pharynx, larynx, and trachea, including regions that liquid reflux struggles to access (90).

Although reflux gas does not directly damage the pharyngeal mucosa, it can serve as a carrier for various active substances, including pepsin, bile acids, and pancreatic enzymes (91, 92). The ability of reflux gas to convey these substances is closely linked to the role of aerosols. When gastric gas refluxes into the pharynx, its high-velocity airflow can fragment gastric juice into aerosol particles, which are subsequently transported by the airflow to the larynx, trachea, and even the lower respiratory tract (92). Thus, gas reflux acts not only as the driving force behind aerosol generation but also as a critical medium for the transport of active substances contained within aerosols to distant sites. The synergistic interaction of these two factors constitutes a significant pathway for distal airway injury in patients with LPR.

Clinical studies indicate a strong association between reflux events and the body's hypoxic state. In a study involving continuous blood oxygen monitoring of 265 patients with LPR, researchers observed that patients experienced a significant decrease in blood oxygen saturation after lying supine for 20 min, with reflux frequency identified as a significant variable influencing this decline in blood oxygen levels (93). In theory, this hypoxic environment may activate hypoxia-inducible factor-1*α* (HIF-1*α*). Based on evidence from other hypoxia-related fibrotic diseases, such as models of peritoneal fibrosis, HIF-1*α* may upregulate the expression of pro-inflammatory and pro-fibrotic genes (94, 95). However, this pathway has not yet been directly validated in the pharyngeal mucosa of LPR patients and remains purely speculative at this time.

In conclusion, reflux gas may hypothetically serve as a transport vector for non-acidic reflux-related substances. Clinical observational data show associations between reflux episodes and episodes of blood-oxygen desaturation. It is hypothesized that reflux-related hypoxia could trigger HIF-1*α* signaling and subsequently up-regulate pro-inflammatory and pro-fibrotic gene profiles, potentially influencing mucosal injury and repair responses. It must be noted that the link between reflux-associated hypoxia, HIF-1*α* activation, and mucosal injury in LPR is entirely inferential. No study has directly demonstrated HIF-1*α* activation in LPR pharyngeal mucosa. The proposed hypoxia-mediated pathway remains speculative and requires direct experimental verification in LPR patients.

## 5 Diagnostic challenges and precise identification tools

The clinical manifestations of LPR exhibit considerable heterogeneity and lack specificity, rendering diagnosis based solely on symptoms and signs significantly constrained. While traditional diagnostic methods are commonly employed in clinical settings, they are characterized by insufficient sensitivity and specificity. In recent years, advancements in the understanding of non-acid reflux have led to the emergence of objective reflux monitoring technology as a central approach for diagnosing LPR (96).

### 5.1 Reflux symptom index and reflux test score

The Reflux Symptom Index (RSI) and the reflux test score (RFS) are currently the most widely utilized clinical assessment tools (97). The RSI comprises nine symptom items, with a total score exceeding 13 suggesting the potential presence of LPR. The RFS evaluates eight signs observed during laryngoscopy, with a total score exceeding 7 indicating a possible diagnosis of LPR (98). However, the symptoms and signs assessed by these tools are not specific to LPR and exhibit a poor correlation with objective reflux monitoring results. Consequently, they cannot serve as a definitive basis for diagnosis (99).

### 5.2 Twenty-four-hour esophageal pH monitoring

Twenty-four-hour esophageal pH monitoring is a well-established method for diagnosing gastroesophageal reflux disease, allowing for quantitative assessment of acid reflux burden. Its strengths include objectivity and repeatability, as it provides quantitative metrics such as the frequency of reflux episodes and duration of acid exposure (100). However, this technique is limited to detecting acid reflux and is incapable of identifying non-acid reflux or weak acid reflux (101).

### 5.3 24-hour multi-channel intracavity impedance-pH monitoring

Traditional 24-hour esophageal pH monitoring is limited to identifying acid reflux and fails to detect weak acid and non-acid reflux events. This limitation underscores the urgent need for a monitoring technology that can comprehensively assess various reflux components in clinical practice. Consequently, the multichannel intraluminal impedance-pH (MII-pH) monitoring may offer a solution. With advancements in technology, MII-pH represents a widely-utilized monitoring tool for detecting acid, weak-acid and non-acid reflux events (102); nevertheless, its diagnostic performance for definite clinical LPR diagnosis remains subject to ongoing debate (103–105). In addition, MII-pH is highly invasive; it requires the insertion of a long, thin catheter that remains in the esophagus for 24 h, causing significant discomfort and leading to poor tolerance among many patients. Furthermore, the high cost of the equipment and the complexity of the procedure hinder its widespread clinical adoption (106).

### 5.4 Dx-pH

The Dx-pH monitoring system represents an innovative approach to oropharyngeal pH monitoring, specifically designed for the diagnosis of LPR (107). This system offers advantages such as simplicity and minimal invasiveness, making it suitable for both screening and monitoring purposes (108). However, some researchers have noted that the clinical utility of Dx-pH data in informing diagnostic or therapeutic decisions is still debated, particularly in the context of surgical interventions (109). In addition, this technology can only detect acid reflux events and has limited ability to identify non-acid reflux.

### 5.5 96-hour wireless pH monitoring

The 96-hour wireless pH monitoring technique involves the placement of a pH capsule in the esophagus during endoscopy. This method extends the monitoring duration, minimizes discomfort in the nasopharynx, and more accurately reflects physiological conditions (110, 111). However, a significant limitation is its exclusive detection of acid reflux, rendering it incapable of identifying non-acid and weak acid reflux events. Consequently, there is a risk of missed diagnoses in patients with LPR primarily characterized by non-acid reflux. Additionally, the high cost of the equipment and the requirement for endoscopic procedures hinder its widespread clinical adoption (112).

### 5.6 Salivary pepsin detection

Salivary pepsin detection has emerged as a significant non-invasive diagnostic technique in recent years. Its advantages include non-invasiveness, simplicity, rapidity, and repeatability. Some studies indicate that its sensitivity can reach 93.8%, rendering it suitable for screening and monitoring therapeutic effects (113, 114). However, the specificity of salivary pepsin detection is relatively low, which raises concerns about the potential for false positives. This method can confirm that pepsin-containing reflux has occurred recently; however, it cannot differentiate between acidic and non-acidic reflux, nor can it provide quantitative data regarding reflux frequency or height. Furthermore, the detection method has yet to be standardized (115, 116).

In conclusion, the field of LPR diagnosis has progressively transitioned from dependence on non-specific symptoms and signs to a multimodal assessment framework that emphasizes objective reflux monitoring technology. Traditional clinical diagnostic methods exhibit significant limitations. Although technologies such as MII-pH can comprehensively identify various reflux events, their invasiveness and operational thresholds hinder widespread clinical adoption. In contrast, emerging technologies like Dx-pH monitoring, 96-hour wireless pH monitoring, and salivary pepsin detection each present distinct advantages; however, they still exhibit shortcomings in detecting non-acid reflux. Overall, current diagnostic methods have yet to achieve an optimal balance among accuracy, convenience, and clinical accessibility. The existing techniques' capacity to identify non-acid reflux events remains inadequate, presenting a central challenge in the diagnosis and treatment of LPR. Second, there are currently no widely accepted reference standards, and the correlation between different monitoring methods remains poor. There is still debate over whether pharyngeal reflux events should be assessed separately using impedance and pH measurements or evaluated using a comprehensive set of criteria. Furthermore, noninvasive testing methods, such as salivary pepsin, have not yet been standardized, and their correlation with objective reflux monitoring results varies across different studies. Future efforts must focus on unifying diagnostic standards and enhancing the integration of these technologies (as shown in Table 2).

## 6 New comprehensive management strategies for non-acid reflux

Given the preceding discussion regarding non-acid reflux substances, it is evident that simple acid suppression may not fully meet the treatment needs of some LPR patients, suggesting that clinical practice should involve individualized assessments based on the patient's specific type of reflux. Therefore, it is essential to evaluate the specific type of reflux experienced by each patient and transition to a treatment model that incorporates physical barriers, mucosal protection, component antagonism, and lifestyle modifications.

### 6.1 Lifestyle intervention

Lifestyle and dietary interventions serve as the cornerstone of all management strategies for LPR (117). Key components of lifestyle intervention include elevating the head of the bed, fasting for three hours prior to sleep, avoiding high-fat and spicy foods, limiting caffeine and carbonated beverages, and abstaining from smoking and alcohol consumption (118). The 2025 European Clinical Practice Guidelines explicitly recommend that empirical treatment should incorporate dietary modifications, stress management, and the use of alginates or antacids to concurrently address both acidic and alkaline reflux events (117). A multicenter randomized controlled trial found that after one month of treatment involving dietary adjustments in conjunction with mucosal protectants, both the RSI and RFS of the patients showed significant improvement, along with a notable decrease in the concentration of pepsin in saliva (119).

### 6.2 Ursodeoxycholic acid

Ursodeoxycholic acid (UDCA) is a class of drugs used to treat bile acid reflux. It functions by displacing and competitively inhibiting toxic hydrophobic bile acids. Additionally, UDCA stabilizes cell membranes, mitigates bile acid-induced oxidative stress, and inhibits the activation of the NF-*κ*B inflammatory pathway. A systematic review has confirmed that UDCA exerts a cell-protective effect in Barrett's esophagus (120). Furthermore, studies indicate that UDCA can alleviate symptoms in patients with bile reflux gastritis by 80% (121). Consequently, for patients with LPR and pathological duodenogastroesophageal reflux, as confirmed by MII-pH monitoring or gastroscopy, the incorporation of UDCA may represent an option when conventional treatments prove ineffective. However, it is important to emphasize that the existing evidence is primarily derived from studies on bile reflux gastritis and Barrett's esophagus, which represent indirect evidence. UDCA has never been studied in LPR patients. The proposed application of this therapy for LPR is based entirely on inferences drawn from other diseases. Consequently, UDCA should not be regarded as a standard or guideline-supported therapy for LPR. High-quality LPR-specific randomized controlled trials are urgently needed before any recommendation for UDCA in LPR can be made.

### 6.3 Alginate

Alginate can form low-density gel rafts in the acidic environment of the stomach, where they float on the surface of gastric contents. As a pH-independent physical barrier, alginate effectively obstructs the upward migration of various reflux products, including pepsin, bile acids, and pancreatic enzymes (122, 123). Tsilivigkos et al. conducted a review of 16 studies encompassing a total of 994 patients, confirming that alginic acid, whether used as monotherapy or as an adjunct to other treatments, provides significant benefits in LPR, particularly in alleviating symptoms associated with non-acid reflux (124). Furthermore, the 2025 European guidelines recommend alginate or antacids, in conjunction with dietary modifications, as fundamental regimens for empirical treatment (125).

### 6.4 Sucralfate

Sucralfate and other mucosal protectants can create a protective gel film on the surface of damaged pharyngeal mucosa. This film physically isolates the mucosa from direct contact with pepsin and bile acids present in the refluxate, thereby mitigating tissue damage (119). Zentilin et al. systematically summarized the mechanisms by which mucosal protectants function, highlighting that sucralfate, alginate-based medications, and novel medical devices can enhance the defense of the esophageal mucosa. These options serve as effective alternatives for patients who exhibit partial or no response to PPI therapy (126). Its function does not depend on acid suppression and can theoretically serve as an adjunctive approach for managing non-acid reflux. However, high-quality clinical studies involving LPR patients are still lacking, and the precise therapeutic effect requires further validation.

### 6.5 Pepsin inhibitors

Based on current research, pepsin inhibitors may be considered a potential treatment option (92, 127). This area has garnered significant attention in recent years. As a contributing factor in non-acid reflux, pepsin's inhibitors may mitigate its proteolytic and inflammatory effects on the pharyngeal mucosa at the source (128). Sales (129) et al. utilized a surgically induced GERD mouse model and observed that both pepstatin (gastrostatin, 0.3 mg/kg) and darunavir (8.6 mg/kg) effectively reduced laryngeal inflammation and preserved the integrity of the laryngeal mucosa after three days of administration. This study indicates that the administration of pepstatin and darunavir can alleviate inflammation and protect the laryngeal mucosa in GERD mouse models. However, there remains a deficit in clinical translational research. Research on pepsin inhibitors remains in the preclinical animal testing phase. Advancing translational research, which involves transitioning from the laboratory to clinical application, is the next critical step. Until this progress is achieved, caution is warranted regarding the rapid movement toward clinical application.

In conclusion, the treatment of non-acid reflux should transition to an integrated model that incorporates physical barriers, component antagonism, mucosal protection, and lifestyle modifications (as shown in Table 3). Currently, there is a paucity of pharmacological studies focused on LPR patients with non-acid reflux, highlighting a significant avenue for future targeted research and therapeutic development (as shown in Table 4).

## 7 Conclusion and prospect

Non-acid reflux may contribute to the pathophysiological process of LPR through multiple mechanisms, including bile acids, pancreatic enzymes, pepsin, and gas reflux; however, its exact clinical causal relationship remains to be further verified. The mechanistic pathways discussed in this review should be viewed as hypotheses requiring rigorous testing in LPR-specific human studies, rather than as established pathophysiological facts. Currently, and there is a notable absence of convenient and precise monitoring technologies for non-acid reflux, as well as a lack of safe and effective biomarkers. Moreover, the existing evidence remains insufficient to establish a clear causal relationship within specific clinical contexts, complicating the determination of optimal intervention timing and targets. The field of non-acid reflux is at a pivotal juncture, transitioning from mechanistic exploration to precision medicine. By prioritizing the generation of high-quality evidence in forthcoming clinical contexts and developing targeted intervention tools and predictive models, it is anticipated that the limitations of current single acid suppression strategies will be overcome, facilitating early warning, precise classification, and comprehensive management of reflux pharyngitis.
